# Analysis of Anxiety Disorders and Post-Traumatic Stress Disorders for Screening Anxiolytic Drugs and Linking Preclinical and Clinical Research

**DOI:** 10.3390/ijms26041414

**Published:** 2025-02-07

**Authors:** Anna Kozłowska, Wan-Jiun Ma, Bai-Chuang Shyu, Andrew Chih Wei Huang

**Affiliations:** 1 Department of Human Physiology and Pathophysiology, Collegium Medicum, University of Warmia and Mazury, Warszawska Av, 30, 10-082 Olsztyn, Poland; kozlowska.anna@uwm.edu.pl; 2 Department of Psychology, Fo Guang University, Yilan County 26247, Taiwan; junusa903@gmail.com; 3 Institute of Biomedical Sciences, Academia Sinica, Taipei 11529, Taiwan; bmbai@gate.sinica.edu.tw

**Keywords:** animal model, anxiety disorders, anxiolytic drugs, post-traumatic stress disorders, preclinical and clinical research

## Abstract

How to determine suitable animal models of anxiety disorders and post-traumatic stress disorders (PTSD) for screening anxiolytic drugs and linking preclinical and clinical research is a crucial issue. This review paper provides background knowledge and critical determination to the animal models for discussing this issue. Moreover, this article analyzes the characteristics, properties, advantages, and disadvantages of various animal models of anxiety disorders and PTSD. It offers an overview of the pathophysiology, treatments, prevalence, and symptoms of anxiety disorders in the clinics. Furthermore, it comprehensively discusses pharmacological treatments and neural mechanisms, as well as the types and properties of the animal models of anxiety disorders in shaping and testing anxiety behaviors. In light of the previous literature discussion, we can understand the different functions of the animal models of anxiety disorders and PTSD to help us link preclinical and clinical research. Animal models are used in advanced drug discovery programs, specifically in psychiatry and neuroscience research. The challenge for the future will be to keep pace with developing the appropriate animal models of anxiolytic drugs to improve the translation of large datasets obtained to clinics.

## 1. Introduction

To our knowledge, a variety of animal models of anxiety disorders and post-traumatic stress disorder (PTSD) have been developed in previous studies to apply in the novel anxiolytic drug screening test and brain mechanism research [1,2]. Therefore, it provides a lot of choices for tests in the preclinical studies and determines the treatment effects of novel anxiolytic drugs in clinics. However, some critical questions have never been evaluated in these animal models. For example, was the chosen animal model more effective than the others? Or whether other animal models were more suitable than the one they determined? This review paper discusses these relevant issues in the following sections to respond to this shortcoming.

Depending on the diagnosis of the Diagnostic and Statistical Manual of Mental Disorders (DSM), anxiety disorders are characterized by excessive fear, anxiety, and related disturbances [3]. Anxiety disorders present heterogenous categories that include generalized anxiety disorder, panic disorder, agoraphobia, conduct disorder, social anxiety disorder (SAD), illness anxiety disorder, bereavement, separation anxiety disorder, selective mutism, obsessive–compulsive disorder (OCD), trauma- and stressor-related disorders, PTSD, and acute stress disorder (ASD) [3,4,5,6]. Although the nosology of anxiety disorders reveals a phenotypical discrepancy in the human model, it is difficult to dissociate the biomarkers and behavioral mechanisms from the various anxiety disorders in the animal model [7,8]. Thus, it is challenging to determine the animal models most appropriate for evaluating the neural mechanisms.

Accordingly, the role of an animal model of anxiety disorders offers the potential for screening current and developing novel anxiolytic drugs [9,10]. Moreover, the most suitable and appropriate animal models are effectively utilized in understanding the behavioral and neural mechanisms of anxiety disorders via anxiogenic and anxiolytic drugs to modulate their symptoms [9,10], and the present findings can be applied in the development of novel anxiolytic medicines. Therefore, the determination of animal models of anxiety disorder is key to novel pharmacological treatments. However, it appears that researchers often do not know how to choose the validated and most appropriate animal models for specific anxiety disorders in the experimental procedures or consider the sexual differences, in part because they do not always understand the advantages and disadvantages of each model [9,10]. Furthermore, researchers may struggle to determine which model has superior reliability and validity to fit their experimental purposes and link with the human models in the clinic.

The review paper addresses the abovementioned issues and offers some insights and implications for selecting ideal animal models of anxiety disorders. First, we comprehensively introduce the prevalence, symptoms, pathophysiology, and treatments of anxiety disorders, including generalized anxiety disorders (GAD), panic disorder, agoraphobia, PTSD, SAD, ASD, separation anxiety disorder, and OCD [3,11]. Although OCD, PTSD, and ASD are excluded from the conventional anxiety disorders in DSM-5 [3], this review paper still discusses OCD, PTSD, and ASD. Second, this study examines numerous anxiolytic drugs and their mechanisms in the brain. For example, common anxiolytic drugs are always involved in the brain GABA system [9,12] or contribute to selective serotonin reuptake inhibitors (SSRIs) via the brain serotonin system [13,14]. Therefore, these distinct mechanisms may help alleviate anxiety disorder symptoms. Third, it also examined anxiolytic substances related to nonclassical neurotransmitters that act via other systems (besides classical neurotransmitters or neuropeptides) to alleviate anxiety symptoms. For example, compounds such as cannabidiol [15] and melatonin [16] were found to reduce the behaviors of anxiety disorders in animal models. Fourth and finally, this review paper analyzes the characteristics, properties, advantages, and disadvantages of various animal models of anxiety disorders, providing some suggestions as to how and when to use these animal models to build up or test anxiety disorders and PTSD, which animal models and test anxiety disorders are best for testing compounds with a specific mechanism of action.

In conclusion, this review paper aims to offer some novelty, including background knowledge and critical determination for screening anxiolytic drug discovery. Moreover, it can promote linking the animal model of anxiety disorders in preclinical research and clinical human studies.

## 2. Previous Literature Selection Methods

This review inquiry uses the PubMed database via keywords to select relevant papers in four sections, including (1) introduction, (2) characteristics, pathophysiology, and treatments of anxiety disorders in clinics, (3) anxiolytic substances used in non-clinical studies, and (4) types and properties for animal models of anxiety disorders. In the Introduction section, 110 potentially relevant papers were identified. After reviewing the title and abstract, 16 articles were assessed for eligibility and included. The characteristics, pathophysiology, and treatments of anxiety disorders in the clinic’s section were selected for 296 potentially relevant papers. A total of 14 papers were eligible and included based on reviewing the title and abstract.

**Figure 1:**
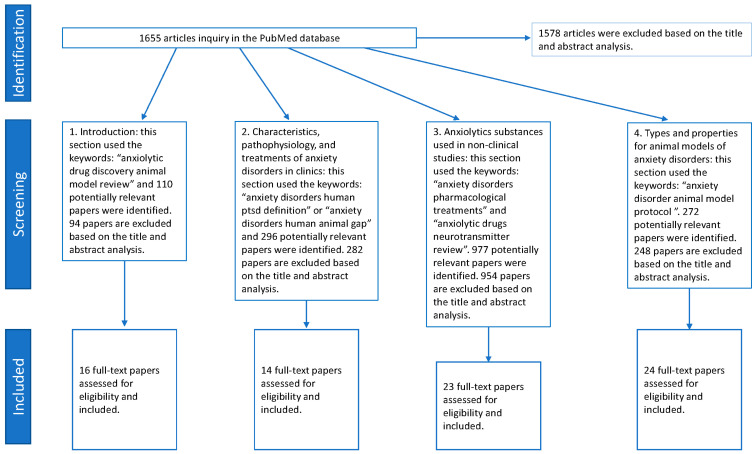
Flow diagram of the article selection process from PubMed database.

In the anxiolytic substances used in the non-clinical studies section, 977 potentially relevant papers were identified. A total of 23 papers were included based on the title and abstract. In the types and properties for animal models of anxiety disorders section, 272 relevant papers were identified. A total of 24 papers were included in this section. In total, 1655 articles were inquired in the PubMed database, and 1578 were excluded (Figure 1).

## 3. Characteristics, Pathophysiology, and Treatments of Anxiety Disorders in the Clinic

### 3.1. Prevalence and Symptoms of Various Anxiety Disorders in the Clinic

In 2017, the World Health Organization reported that the total estimated number of people with anxiety disorders in the world was 264 million [17]. Moreover, it was separated by regions and showed the prevalence of anxiety disorders. For example, the African region was 10%; the Eastern Mediterranean region was 12%; the European region was 14%; the region of the Americas was 21%; the South-East Asia region was 23%; and the Western Pacific region was 20% [17]. According to the DSM-5 descriptions of anxiety disorders in clinical aspects, the essential symptoms of many anxiety disorders vary widely [3,11]; anxiety disorders are shown to be heterogeneous and exhibit diverse phenotypes in humans [4,11]. For example, the major anxiety disorders can be separated into eight phenotypical categories. The present and discussed anxiety disorders include GAD, panic disorder, agoraphobia, PTSD, SAD (i.e., social phobia), ASD, separation anxiety disorder, and OCD.

According to the DSM-5 [3], the major symptoms of GAD are chronic, excessive anxiety and worry about various domains, including school and work performance. GAD has a 12-month prevalence of 0.9–2.9% in adults and adolescents in the United States [3]. The major symptoms of panic disorder include recurrent unexpected panic attacks, and individuals with panic disorder are persistently concerned with and worried about further panic attacks, potentially changing their behaviors in maladaptive ways [11]. Panic disorder has a 2–3% prevalence rate for adolescents and adults in the United States [3]. The major symptoms of agoraphobia are intense fear or anxiety induced by an extendable range of surroundings in real or anticipated exposure; the 12-month prevalence of agoraphobia is nearly 1.7% for adolescents and adults in the United States [3]. The major symptoms of PTSD include intrusions and avoidance of memories associated with traumatic events. The critical features of PTSD vary [18]. Some patients encounter fear-based reexperiencing, emotional, and behavioral symptoms. Others feel anhedonic or dysphoric mood states, and negative cognitions may be most distressing. In some cases, arousal and reactive-externalizing symptoms are prominent; however, others produce dissociative symptoms that predominate. Particularly, some individuals exhibit combinations of these symptom patterns. The 12-month prevalence of PTSD is 3.5% in the United States [3]. The major symptoms of SAD include fear and anxiety about, or avoidance of, social interactions and social surroundings when they involve the possibility of being scrutinized [18]; the 12-month prevalence of SAD is approximately 7% in the United States [3]. The major symptoms of ASD, which follow one or more traumatic events, include the development of anxiety lasting from three days to one month after the event(s) [3]. The prevalence of ASD is less than 20% (that does not involve interpersonal assault) in the United States [3]. The critical features of separation anxiety disorder are excessive fear or anxiety concerning separation from home or attachment figures [11]. The 12-month prevalence of separation anxiety disorder in the United States is about 0.9–1.9% for adults, 4% in children, and 1.6% in adolescents [3]. The major symptoms of OCD are, as the name implies, obsessions and compulsions; obsessions are chronic and repetitive thoughts, images, or urges, whereas compulsions are persistent behaviors and mental acts [19]. The 12-month prevalence of OCD is about 1.2% in the United States [3]. In descending order of prevalence among adults, the three most common of these eight anxiety disorders are ASD, SAD, and PTSD. The diversity of the eight disorders’ major symptoms is shown in Table 1. 

### 3.2. Pathophysiology of Anxiety Disorders in the Clinic

A growing body of evidence showed that the dorsomedial prefrontal cortex, insula, and amygdala were involved in anxiety disorders [20,21,22,23]. The threat circuit concerning the projections between the dorsomedial prefrontal cortex and amygdala induces the activation and coupling of the dorsomedial prefrontal cortex-amygdala circuit for healthy people when they experience fear, threat, and aversive stimuli [24]; however, anxiety disorders enhance the activations in the dorsomedial prefrontal cortex-amygdala circuit when exposed to the situation related to the danger and aversive stimuli [24]. Threat stimuli are positively associated with coupling the dorsomedial prefrontal cortex and amygdala in hyperactivation [21,22]. Therefore, the dysfunction of the dorsomedial prefrontal cortex-amygdala circuit contributes to the pathophysiology of various anxiety disorders.

### 3.3. Pharmacological Treatments of Anxiety Disorders in the Clinic

It is a crucial issue how to lessen the hyperactivity and coupling of the dorsomedial prefrontal cortex-amygdala circuit, leading to severe fear and vigilance symptoms of anxiety disorders. To date, numerous pharmacological treatments have been employed in clinics for eight major types of anxiety disorders in DSM-5 (Table 2). For example, benzodiazepines (BDZs) are the GABAa receptor agonists that are underlined to inhibit the activity of postsynaptic neurons for alleviating symptoms of anxiety disorders [25]. In the clinic, almost all BDZ drugs showed non-specific anxiolytic-like effects, and all BDZs can treat all anxiety disorders, such as GAD, PD, agoraphobia, PTSD, SAD, ASD, separation anxiety disorder, and OCD; only clonazepam was used in PD but no other anxiety disorders (Table 2) [25]. SSRIs have been shown to reduce the hyperactivity of the dorsomedial prefrontal cortex-amygdala circuit, resulting in the amelioration of a variety of anxiety disorders [26]. In clinical findings, GAD can be alleviated by escitalopram, paroxetine, and sertraline [24]. The behavioral symptoms of PD and agoraphobia are alleviated by escitalopram, fluoxetine, fluvoxamine, paroxetine, citalopram, and sertraline [27]. Paroxetine and sertraline treatments alleviated PTSD’s symptoms [25]. The anxiety symptoms of SAD could be effectively reduced by escitalopram, fluvoxamine, paroxetine, citalopram, and sertraline [27]. The clinical studies of OCD showed that fluoxetine, fluvoxamine, paroxetine, and sertraline could decrease the symptoms of OCD [25]. Notably, any drugs of SSRIs cannot effectively treat the symptoms of ASD and separation anxiety disorder (Table 2). Serotonin-norepinephrine reuptake inhibitors (SNRIs) are another kind of anxiolytic drug. A previous study demonstrated that SNRIs interacted with enzymes that produce enzyme inhibitions, and then alleviated anxiety symptoms, reducing the activation of the dorsomedial prefrontal cortex-amygdala circuit [28]. Duloxetine treats GAD symptoms; however, venlafaxine treatments can reduce GAD, PD, agoraphobia, and SAD (Table 2). Tricyclic antidepressant (TCA) medicines have been demonstrated to inhibit the reuptake of serotonin and norepinephrine, and they decrease the symptoms of anxiety disorders. Especially for the mechanism of serotonin reuptake inhibitors, the TCA drugs (e.g., clomipramine and doxepine) could reduce OCD symptoms [29]. Clomipramine could decrease PD, agoraphobia, and OCD; doxepine ameliorated almost all anxiety disorders, including GAD, PD, agoraphobia, PTSD, SAD, ASD, separation anxiety disorder, and OCD; imipramine only treated PD patients (Table 2). Monoamine oxidase inhibitors (MAOIs) act to inhibit the activity of monoamine oxidase within neurons. Then, it reserved higher amounts of monoamine neurotransmitters (e.g., dopamine, norepinephrine, epinephrine, and serotonin) in the presynaptic neurons [30]. In MAOIs, phenelzine can treat PD disorders; moclobemide can ameliorate the SAD symptoms (Table 2). Pregabalin, a kind of calcium modulator, was found to act at the alpha-2-delta subunit of voltage-dependent calcium ion channels for alleviating the anxiety symptoms of GAD and SAD (Table 2). A 5-HT_1A_ agonist, buspirone, and azapirone can effectively relieve anxiety symptoms for non-specific anxiety disorders; antihistamine hydroxyzine affiliates with histamine 1 receptors to reduce non-specific anxiety behaviors [25]. In conclusion, current anxiolytic drugs in the clinic go through different mechanisms to reduce the hyperactivity and coupling of the dorsomedial prefrontal cortex-amygdala circuit for amelioration of anxiety symptoms of anxiety disorders. 

## 4. Anxiolytic Substances Used in Non-Clinical Studies: Pharmacological Treatments and Neural Mechanisms

### 4.1. Conventional Anxiolytic Substances

In the clinics, anxiolytic drugs are applied in patients with a variety of anxiety disorders; however, the same compounds are employed and tested for anxiety behaviors or anxiety activity in the animal model, which is called anxiolytic substances (but not anxiolytic drugs). This section introduces numerous anxiolytic substances to reduce anxiety activity in animal models. To our knowledge, anxiolytic substances include the categories of classical neurotransmitters, neuropeptides, and nonclassical neurotransmitters; moreover, nonclassical neurotransmitters are currently concerned and considered for developing novel medicines in anxiolytic drug discovery. As mentioned in the clinic above, the dorsomedial prefrontal cortex-amygdala hyperactivity and coupling are the essential pathophysiology for a variety of anxiety disorders [20,21,22,23]. The crucial issue is alleviating the dysfunction of the dorsomedial prefrontal cortex-amygdala circuit, which results in an anxiolytic-like effect [20,21,22,23]. This section introduces how the researcher uses animal models and a variety of anxiolytic substances to underline different neural mechanisms to reduce the dysfunction of the dorsomedial prefrontal cortex-amygdala circuit and to evaluate anxiety disorders (Table 3). For example, common anxiolytic substances that interact with classical neurotransmitter systems include benzodiazepines (BDZs) and SSRIs, as well as dopamine-, norepinephrine-, NMDAR-, and histamine-related drugs. In the animal model of anxiety disorders, such as conditioning fear learning or PTSD, the neutral stimulus (i.e., conditioned stimulus) paired with aversive or threat events (i.e., unconditioned stimulus) to form the fear conditioning, and then it produces anxiety behaviors [9]. Treatment plans with BDZs are often applied in the conditioned fear learning paradigm of the animal model to assess anxiety disorders and PTSD; moreover, BDZs affiliated with GABAa receptors lead to an influx of chloride ions into the neuronal membranes, thereby inhibiting postsynaptic potentials and inducing anxiolytic-like effects [31,32]. SSRIs have been applied in cue or contextually conditioned fear learning and PTSD animal models to mimic multiple anxiety-related disorders; SSRIs inhibit serotonin reuptake and increase serotonin levels in the synaptic cleft to cause anxiolytic-like effects [14,33]. Dopamine systems could be modulated using pharmacological tools to test the single prolonged stress (SPS) animal model of PTSD; it was found that D2/D3 agonists could disrupt anxiety disorders through D2/D3 receptors [34]. Some studies have examined how the norepinephrine system modulates anxiety disorders and PTSD symptoms, demonstrating that alpha-1 adrenergic receptor antagonists blunted anxiety- and PTSD-associated fear behaviors, thereby inducing anxiolytic-like effects [35,36]. NMDA receptor (NMDAR) functions were associated with anxiety disorders and PTSD; the animal study of conditioned fear learning showed that promoting NMDAR functions decreased fear symptoms [37]. Moreover, the NMDAR antagonist impaired fear extinction, whereas the NMDAR agonist facilitated the consolidation of fear extinction [37]. Histamine, which may affiliate with H3 receptors, has been shown to reduce anxiety disorders in the animal model of conditioned fear learning and isolation-induced aggressive behaviors [38]. In summary, classical neurotransmitter systems such as those cited above could modulate a range of anxiety disorders.

In contrast to the classical neurotransmitter systems, the involvement of neuropeptide systems in anxiety disorders and PTSD was relatively less common [39,40,41]. For example, previous studies showed that morphine (i.e., mu-opioid receptor agonist) attenuated PTSD symptoms in the conditioned fear learning paradigm [41,42]. Neuropeptide Y reduced anxiety behaviors and PTSD symptoms via neuropeptide Y receptors in the single-prolonged stress PTSD animal model, indicating that neuropeptide Y receptors are likely another potential mechanism for the modulation of anxiety disorders [40]. Orexin neurons have multiple functions for regulating physiological responses such as feeding, reward, and thermogenesis [43,44]; some recent studies have demonstrated that the blockade of orexin receptors may impair fear behaviors and enhance the consolidation of fear extinction [39].

Aside from the modulation of classical neurotransmitters and neuropeptide systems in anxiety disorders and PTSD, anxiolytic drugs related to nonclassical neurotransmitters were found to make essential contributions in alleviating anxiety disorders and PTSD symptoms via diverse neural mechanisms in preclinical and clinical trials. For example, pro-inflammatory cytokines can impair signaling involved in reward and the modulation of fear/anxiety, causing anxiety disorders and PTSD symptoms; inflammatory cytokines can be considered for developing novel therapeutic strategies to reduce anxiety disorders and PTSD [45]. Brain-derived neurotrophic factor (BDNF) is a protein, a member of the neurotrophin family of growth factors, and has neuroprotective roles in neuronal growth; BDNF has been shown to alleviate PTSD symptoms via the BDNF-TrkB signaling pathway within neurons using the SPS PTSD animal model [46,47]. Research on glucocorticoids underlined that the hypothalamus–pituitary–adrenal gland system interferes with PTSD symptoms in the PTSD animal model [48]. Melatonin impaired contextual fear conditioning via MT1 and MT2 receptors, resulting in the amelioration of PTSD symptoms [16]. Animal models have indicated that CBD may have the potential to impair multiple anxiety disorders (e.g., generalized anxiety disorder, panic disorder, SAD, PTSD) through CB1 receptors [15]. Other studies have focused on the relationship between the blockade of ion channels and anxiety disorders and PTSD symptoms. For example, L-type calcium channel blockers such as lacidipine interfere with caffeine-induced anxiety symptoms by blocking calcium channels, thus producing anxiolytic-like effects [49]. Sodium channel blockers such as lamotrigine appear to reduce anxiety symptoms via sodium channel inhibition in the cue-conditioned fear learning paradigm [50]. In conclusion, these anxiolytic substances related to nonclassical neurotransmitters, which act through diverse neural mechanisms, have been shown to attain anxiolytic-like effects in multiple anxiety disorders and PTSD symptoms.

**Table 3:** Comparison of anxiolytic substances and animal models in specific anxiety disorders.

Mechanism of Action	Mental Illness	Animal Models	Neural Mechanisms and Effects	References
Classical Neurotransmitters:				
1. Agonism of GABAa receptor	Anxiety disorders and PTSD	Conditioned fear learning	1. BDZ drugs affiliate with the GABAa receptor 2. Cause anxiolytic effects	[31,32]
2. Inhibition of serotonin reuptake	Anxiety-related disorders (e.g., panic disorder, generalized anxiety disorders, PTSD)	Conditioned fear learning (contextual or cue) or PTSD animal models	1. SSRIs drugs act on the inhibition of serotonin reuptake 2. Lead to anxiolytic effects	[14,33]
3. Agonism of dopamine receptor	PTSD	PTSD animal model (single prolonged stress)	1. D2/D3 receptor agonism 2. Lead to anxiolytic effects	[34]
4. Antagonism of norepinephrine receptor	PTSD	Conditioned fear learning	1. Antagonism of alpha-1 adrenergic receptor 2. Disrupt anxiety- and PTSD-associated symptoms	[35,36]
5. Antagonism of NMDA receptor	Anxiety disorders and PTSD	Conditioned fear learning animal model	1. Antagonism of NMDA receptor 2. Attenuate fear symptoms	[37]
6. Agonism of histamine receptor	Anxiety disorders	Isolation-induced aggressive behavior; conditioned fear learning	1. H3 receptor agonism 2. Reduce anxiety disorders	[38]
Neuropeptides:				
1. Agonism of opiates	PTSD	Conditioned fear learning	1. Opioid receptor agonism 2. Result in anxiolytic effects	[41,42]
2. Activation of neuropeptide Y	PTSD	PTSD animal model (single prolonged stress)	1. Neuropeptide Y receptor agonism 2. Reduce anxiety behaviors and PTSD symptoms	[40]
3. Antagonism of orexins receptor	Anxiety disorders (e.g., phobia, panic, and PTSD)	Conditioned fear learning animal models	1. Orexin receptor antagonism 2. Impair fear behaviors	[39]
Nonclassical neurotransmitters:				
1. Activation of inflammatory cytokines	Anxiety disorders and PTSD	Multiple anxiety and PTSD animal models	1. Activation of inflammation cytokines 2. Cause anxiety disorders and PTSD symptoms.	[45]
2. Activation of BDNF	Anxiety disorders and PTSD	PTSD animal model (single prolonged stress)	1. Activation of BDNF via TrkB receptor 2. Attenuate anxiety disorders	[46,47]
3. Activation of glucocorticoid	PTSD	PTSD animal models	1. Activation of glucocorticoid receptor 2. Block anxiety disorders	[48]
4. Activation of melatonin	PTSD	Conditioned fear learning animal models	1. Activation of melatonin receptor 2. Impairs contextual fear conditioning	[16]
5. Activation of cannabidiol	Anxiety disorders (e.g., generalized anxiety disorder, panic disorder, social anxiety disorder, PTSD)	Multiple anxiety disorder animal models	1. Agonism of the CB1 receptor 2. Impair multiple anxiety disorders (including generalized anxiety disorder, panic disorder, social anxiety disorder, and PTSD)	[15]
6. Action of L-type calcium channel blocker	Anxiety disorders	Caffeine-induced anxiety symptoms	1. Antagonism of calcium channels 2. Cause anxiolytic effects	[49]
7. Activation of sodium channel blocker	PTSD	Conditioned fear learning (i.e., cue)	1. Antagonism of sodium channels 2. Lead to anxiolytic effects	[50]

Note: BDNF: brain-derived neurotrophic factor; PTSD: post-traumatic stress disorder.

### 4.2. Current Anxiolytic Substances: Classical Neurotransmitters, Neuropeptides, and Nonclassical Neurotransmitters

**Figure 2:**
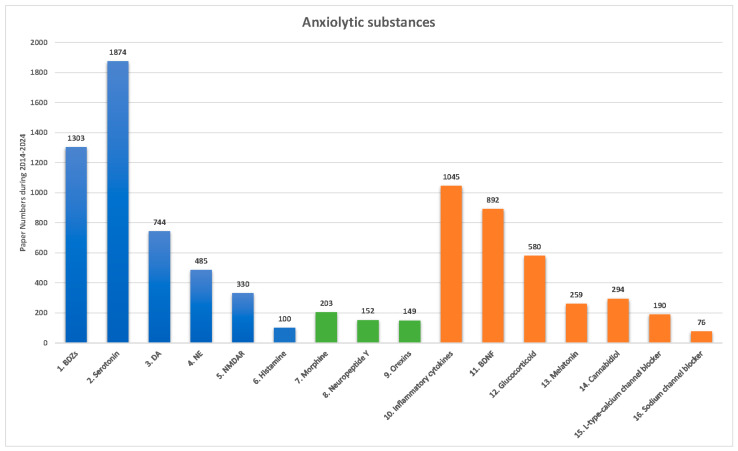
Figure depicts paper numbers during 2014–2024 found by searching the PubMed database for various anxiolytic drugs and substances: BDZs, serotonin, DA, NE, NMDAR, histamine, morphine, neuropeptide Y, orexins, inflammatory cytokines, BDNF, glucocorticoid, melatonin, cannabidiol, L-type calcium channel blocker, and sodium channel blocker. The blue vertical bars are involved in classical neurotransmitter systems. The green vertical bars indicate neuropeptide systems. The orange vertical bars represent the nonclassical neurotransmitter systems. BDZs: benzodiazepines; DA: dopamine; NE: norepinephrine; NMDAR: NMDA receptor; BDNF: brain-derived neurotrophic factor.

Concerning the previous studies related to anxiety disorders and pharmacological treatments, a growing body of evidence has shown that various categories of anxiolytic drugs and substances (including novel drugs) could reduce anxiety disorders and PTSD symptoms [13,27,51,52]. PubMed was searched to gather data on the number of published papers with different keywords for anxiety disorders and specific anxiolytic substances (e.g., BDZs, SSRIs) for 2014–2024 (Figure 2). As Figure 2 depicts, current anxiolytic substances can be separated into classical neurotransmitter systems-related medicines (in blue bars), neuropeptide systems-related medicines (in green bars), and nonclassical neurotransmitter-related compounds (in orange bars).

Considering the clinical findings, patients with depression have an approximate 85% rate with anxiety disorders; however, patients with anxiety disorders have an almost 90% rate to be comorbid with depression [53]. Therefore, anxiety disorders are highly comorbid with depression. Interestingly, SSRI antidepressant drugs were featured in the most published articles, with 1874 papers. The conventional anxiolytic drugs, BDZs, were ranked second, with 1303 papers. These findings indicate that anxiety disorders and major depression disorders seemingly share similar neural mechanisms; moreover, the present evidence was supported by the clinical data that anxiety disorders were found to be comorbid with major depression disorders [53]. Concerning the neural mechanism of anxiolytic disorders, researchers see serotonin systems as offering greater potential than GABA systems, especially during 2014–2024. Inflammation cytokines were ranked third, appearing in 1045 published papers in connection with anxiety disorders. Administering inflammatory cytokines may induce fear, anxiety disorders, and PTSD, which is related to the inflammation system. Accordingly, the inflammation system is an alternative consideration for developing anxiolytic drugs. BDNF was ranked fourth, with 892 published papers. BDNF, a novel treatment for anxiety disorders and PTSD that is currently in the preclinical phase of animal models, goes through the BDNF-TrkB signaling conductions to alleviate anxiety disorders and PTSD symptoms. D2/D3 receptors via the dopamine system were ranked fifth, with 744 published papers; dopamine antagonism may alleviate anxiety disorders and PTSD symptoms. The other anxiolytic substances associated with classical neurotransmitters had fewer published papers, including 485 for norepinephrine, 330 for NMDA, and 100 for histamine. Neuropeptides morphine, neuropeptide Y, and orexins appeared in even fewer papers, reflecting their novelty as pharmacological treatments; very likely, more studies involving them will be published soon. The anxiolytic substances related to nonclassical neurotransmitters, including glucocorticoids, melatonin, cannabidiol, L-type calcium channel blockers, and sodium channel blockers, appeared in the fewest publications, suggesting ample room for their development as novel and promising pharmacological anxiolytic treatments.

## 5. Types and Properties for Animal Models of Anxiety Disorders

A previous study has suggested that the animal models of anxiety disorders could be divided into the models based on unconditioned responses and conditioned responses [54]. However, the present review paper does not follow this division for the animal models of anxiety disorders. Based on experimentation and statistical hypothesis testing, the experimental variables can be divided into independent and dependent variables [55]. The independent variable is defined as the causes of behaviors for individuals or groups; however, the dependent variable is viewed as the effect of behaviors for individuals or groups [55]. Therefore, you manipulate independent variables to affect the outcome of an experiment. Dependent variables represent the outcome of the experiment.

In the present animal models, we define the animal models for testing anxiety disorders as consisting of independent and dependent variables. Independent variables can be manipulated to shape various animal models of anxiety disorders as well as PTSD (i.e., it is like a cause for behaviors). Alternatively, another kind of anxiety disorder in the animal models belongs to the dependent variables (i.e., it is like an effect of behaviors), and these kinds of anxiety behaviors are not able to be manipulated. Instead, animal models of this type can be used to test for various anxiety responses in anxiety disorders and PTSD. Suitable animal models of anxiety disorders and PTSD should be selected based on requirements of face, predictive, and constructive validities [56]. Face validity refers to whether it is obvious on the surface that the tested behaviors and symptoms of the animal model are comparable to the symptoms in humans; predictive validity means that the anxiolytic drugs can effectively alleviate symptoms and behaviors; finally, constructive validity indicates that an anxiety disorder in an animal model shares the same brain mechanism as in humans [57,58]. In addition to considerations of these three validity types, animal models of anxiety disorders and PTSD should be considered in light of their characteristics, advantages, disadvantages, when to use them, and usage frequencies.

### 5.1. Shaping an Animal Model of Anxiety Disorders and PTSD

Determining a suitable and shaping animal model for anxiety disorders and PTSD is a crucial issue. The review paper offers numerous types and properties of developed animal models (see Table 4A: Shaping anxiety models). For example, anxiety disorders and PTSD of fear conditioning were continuously used in building up a reliable and valid animal model. Fear conditioning models of this type can be separated into cue stimulus [59] and contextual stimulus [60] (conditioned stimulus, CS) to pair with footshock-induced stress (unconditioned stimulus, US); thus, the animal models could induce fear behavior to mimic anxiety responses. The cue model has advantages, including clear-cut stimulus as well as excellent face, predictive, and constructive validities [59].

In contrast, the contextual model was designed to apply a complex contextual stimulus that combined different environmental stimuli to pair with footshock stress [60]. Accordingly, the contextual stimulus is very similar to the environmental stimulus. Thus, the contextual model has the advantage of being very similar to natural, environmentally induced PTSD or anxiety disorders. However, because the context is a complex stimulus that is not easy to manipulate, the context itself may bring disadvantages. Regardless of their disadvantages, cue and contextual models were often used in research on anxiety disorders and PTSD. Moreover, both models are used very often in the animal models for PTSD and anxiety disorders.

SPS is another kind of animal model for PTSD [61]. The SPS PTSD animal model has three stages for manipulating PTSD behaviors. For example, the animal should be restrained for 2 h and then forced to swim for 20 min. After recovering for 15 min, animals were exposed to ether until loss of consciousness. The advantages of this model are long-term, stable stress, excellent face, and predictive and constructive validities. Its disadvantages are long-term stress treatments and manipulations because the real condition of PTSD is likely very short-term, and its symptoms are overwhelmingly stressor-induced. Thus, this SPS model of PTSD may not be ideal due to concerns about validity and reliability. Moreover, this model of SPS is firstly considered for application in inducing the symptoms of PTSD in the animal model.

Another animal model used to test anxiety and depression behaviors is learned helplessness [62,63]. This experimental procedure is designed so that animals are exposed to uncontrolled stressors through behavioral responses. The advantages of this model include effective and easy stressor manipulation. However, this model can also be used to test depression behaviors; thus, its findings cannot be differentiated between anxiety and depression tests—this is its chief disadvantage. This model has rather fewer legitimate opportunities for application in anxiety tests.

The restraint stress model [64] induces immobility in mice by placing them into well-ventilated 50 mL Falcon tubes for 2 h per day over 21 consecutive days. This animal model is easy to conduct but is also used to test depression behaviors. Thus, its results cannot be clearly differentiated for anxiety and depression behaviors. Another animal model, inescapable tail shock, is designed to test PTSD fear behavior [65,66]. The animal would experience uncontrolled and inescapable tail shock, causing them acute stress. It is easy to conduct, but because it is also employed in testing depression behaviors, it suffers from the same disadvantages that affect the two previous models, namely the difficulty in differentiating results for anxiety and depression. For the underwater trauma model [67], animals are held underwater for 30 s to induce severe stress. The advantage of this model is that it is easy to manipulate to shape stressors and develop the PTSD model. However, because its face, predictive, and constructive validities remain in doubt, it is seldom used in the animal model of PTSD. Under the social isolation model [13], the animal is raised alone with no companion. This model is easily conducted to produce stress; however, the long-term conduction time is its disadvantage. A related model, social defeat, requires that animals be exposed to a trained aggressor conspecific for 6 h per day for 5 or 10 days [68]; the advantage of this model is easy conduction. Early-life stress is designed to use maternal separation and a trauma event to mimic PTSD symptoms [67]; the advantages of this model are good face, predictive, and constructive validities, but this model requires conducting a chronic procedure of stress, which constitutes its chief disadvantage. Finally, predator-based stress [67] uses predators or predator-related stimuli (such as a predator’s urine) to induce the trauma event; its advantage is the ease of conduction.

### 5.2. Testing Anxiety and PTSD Behaviors

The testing animal models of anxiety disorders and PTSD served as the dependent variables that are different from the previous descriptions of shaping the animal models of anxiety and PTSD (see Table 4B: Testing anxiety behaviors). In these examples, the open field test (OFT) and elevated plus maze test (EPMT) are the most popular models for testing anxiety behaviors.

The OFT measures time spent and crossing trials in the center area of the task [69,70]. Increased (decreased) time spent and more (fewer) crossing trials in the center area indicate lower (higher) anxiety responses. Note that preclinical scientists with expertise in the field of anxiety would not use this test except to control for adverse drug effects. Alternatively, the OFT also tests locomotion activity; thus, its disadvantage is the competition between locomotion and anxiety behaviors, which cannot be clearly differentiated. On the other hand, the time spent by the animal in the central part of the open arena in some situations may result from changes in spontaneous locomotor activity; therefore, the results of this test should be interpreted with caution.

The EPMT, another popular animal model [69], measures time spent in the open arm of a maze to indicate the strength of anxiety responses. The advantages of the EPMT are that the animals can rest in the crossing area between the open and closed arms. However, the disadvantages of the EPMT are that the animal sometimes stays longer in the crossing area, leading to errors of measurement for anxiety behaviors. The elevated zero maze and x-maze tests are similar to the EPMT, albeit with different shapes. The elevated zero maze test is designed in the shape of a circle with two sets of open arms and two sets of closed arms; however, it lacks the crossing area [68]. Thus, the advantage of the elevated zero maze test is its lack of a crossing area, which enforces animals’ decisions. However, conflicts arise from time spent in the open and closed arms; the elevated x-maze task tests the open arm time and total time ratio. Its face, predictive, and constructive validities are good [71,72]. The light–dark box test assesses activity and time spent in the brightly lit and dark apparatus compartments based on the animals’ innate desire to explore novel areas [69]; the advantage is easy conduction. The startle response test pairs a conditioned stimulus with a footshock to induce startle responses that serve as anxiety responses [10]. This model of startle responses has good face, predictive, and constructive validities for anxiety disorders; however, this model is limited to the anxiety behaviors related to a cue with footshock conditioning. In the marble burying test of anxiety responses, animals with previous stress are placed in the test cage; the depth of marble burying is tested up to 2/3 of the depth with bedding [73]. It appears to offer excellent face, predictive, and constructive validities for anxiety disorders; however, because digging behavior is a species-typical reaction to stress, it should be considered for use only in the specific species of animals that are prone to such behavior. The defensive shock-prod burying test is designed such that animals encounter an electrical probe connected to a shock source, and then it measures the depth to which the prod is buried [74]. Face, predictive, and constructive validities are good for anxiety disorders in the defensive shock-prod burying test; however, sometimes, animals do not touch the electrical probe, which thus cannot induce anxiety behaviors. The grooming test uses stressors to induce grooming behaviors, including novel environments, predator exposure, and bright light [10]. Simple conduction is its advantage; however, it has questionable face, predictive, and constructive validities. The social interaction test is designed such that two animals are placed in the test environment for 5 or 10 min while the duration and frequency of all social interactions—including sniffing, following, chasing, touching, and biting—are recorded [10]; higher scores indicate lower anxiety behaviors. Accessible design and easy conduction are its advantages; the disadvantages are limited applicability to SAD. The Suok task simultaneously measures anxiety, vestibular, and neuromuscular deficits through an unstable rod with novelty [75]; the threats of height, loss of balance, and novelty are presented to analyze anxiety and assess animal exploration. This model may have face validity; however, competition among several behaviors has occurred in the Suok test, constituting a notable disadvantage. The stress-induced hyperthermia test is based on the evolutionarily important role of hyperthermia, which increases body temperature in response to encountering stressors [10]. This model is applicable across many species, including humans. Its disadvantages are due to testing errors from numerous confounding factors. The hole-board test assesses head-dipping behaviors [76]; more head dips represent less anxiety and more explorations. This model enjoys easy preparation and conduction; however, there are doubts about its face, predictive, and constructive validities. The rat exposure test is dependent on the animals’ natural defensive “avoidance” behavioral responses to signs of potential danger [10]; defensive behaviors include stretch-attend posture, stretch approach, freezing, burying, and hiding. This model enjoys easy conduction; however, there are variances for different species in the present model. The novel object test measures the approach-avoidance behaviors of the animals in response to novel stimuli [77]; longer spent time in exploration for a novel object indicates lower anxiety behaviors. This model has excellent face, predictive, and constructive validities. However, the disadvantages are that this model is also applied in the recognition function, and it is confused with recognition tests.

In conclusion, animal models have different characteristics, advantages, and disadvantages. In the following section, we provide some suggestions as to how and when to use these animal models to build up or test anxiety disorders and PTSD. Face, predictive, and constructive validities should be basic considerations when selecting the most appropriate model for the determinations.

## 6. Opinion from Preclinical Studies to Clinical Research

This review paper sought background knowledge and critical determination for screening anxiolytic drug discovery. Moreover, it can help narrow the gaps between the animal model of anxiety disorders in preclinical research and clinical human studies. Choosing the most appropriate animal model of anxiety disorders is crucial because the best animal models could precisely target specific anxiety disorders in humans. In addition, the most suitable animal model could be fully explained and applied to anxiety disorders in clinical settings.

To summarize the information in the above tables, the animal models of fear conditioning with cue and learned helplessness models can be used for testing the brain mechanism of GAD and clinical drugs, including BDZs, SSRIs, SNRIs, TCAs, calcium modulators, azapirones, and antihistamines. Fear conditioning with context, inescapable tail shock, and underwater trauma are the most appropriate animal models of anxiety disorders for testing PD with BDZs, SSRIs, SNRIs, TCAs, MAOIs, azapirones, and antihistamines in the clinics. Agoraphobia was tested in fear conditioning with context and assessing anxiolytic drugs such as BDZs, SSRIs, SNRIs, TCAs, azapirones, and antihistamines. PTSD can be tested by numerous animal models, including fear conditioning with cue or context, SPS, learned helplessness, restraint stress, inescapable tail shock, underwater trauma, social isolation, social defeat, early-life stress, and predator-based stress; moreover, BDZs, SSRIs, SNRIs, TCAs, azapirones, and antihistamines can be tested by the mentioned models. Because of social properties, SAD was suggested using social isolation and social defeat to test BDZs, SSRIs, SNRIs, TCAs, MAOIs, calcium modulators, azapirones, and antihistamines. ASD has the properties of short-term and severe stress, and thus, fear conditioning with cue or context, restraint stress, inescapable tail shock, and predator-based stress for testing BDZs, TCAs, azapirones, and antihistamines. Separation anxiety disorder can be tested by the animal models of anxiety disorders, including social isolation and early-life stress, because of the social separation effect; moreover, BDZs, TCAs, azapirones, and antihistamines can be tested by these animal models. OCD was suggested to use fear conditioning with the cue, learned helplessness, inescapable tail shock, and predator-based stress for tests in clinical drugs such as BDZs, SSRIs, TCAs, azapirones, and antihistamines (Table 5).

Current anxiolytic substances interact with different neural mechanisms, including classical neurotransmitter, neuropeptide, and nonclassical neurotransmitter systems. The present developmental lines of anxiolytic substances may potentially attenuate multiple anxiety disorders and PTSD. The most suitable anxiolytic substances can be identified for the amelioration of multiple anxiety disorders. SSRIs were the most referenced in clinical and preclinical aspects of the current anxiolytic substances related to classical neurotransmitter systems. The conventional anxiolytic substances, BDZs, were ranked second. Anxiolytic substances related to neuropeptides may be considered for the development of novel pharmacological treatments. The nonclassical neurotransmitters (except for inflammatory cytokines and BDNF) also appeared in fewer published papers. This line of studies needs to be examined in the future.

On the other hand, multitargeted drugs can be considered to alleviate the symptoms of anxiety disorders. For example, the novel treatment combined the first three priority anxiolytic drugs, BDZ, SSRI, and anti-inflammatory cytokines. The mixed drugs might produce the optimal amelioration effects of anxiety disorders. This issue should be investigated in further study.

In summary, some suggestions can be provided on how and when to use these animal models to build up or test anxiety disorders and PTSD. For example, when the researcher wants to manipulate a stressor to induce the animal model of anxiety disorders, the researcher can use “the shaping anxiety models”. Moreover, the fear conditioning in the cue (or context) pairing with footshock or the SPS PTSD model is the appropriate animal model of anxiety disorders and PTSD. On the other hand, when the researcher wants to test anxiety behaviors, they can use the open field test or elevated plus maze test. The face, predictive, and constructive validities should be basic requirements for considering which model is most apt for the determinations. 

## 7. Limitations

Some limitations emerged in the present review. First, the present review is a narrative review paper. Thus, there might be selection bias in the previous literature. We suggested that the systematic and meta-analysis reviews were more objective in elucidating the same issues. Further review papers should consider the probable shortages. Second, some animal models of anxiety disorders and PTSD were easily mixed uses for screening the novel anxiolytic drugs. However, we need to consider developing novel animal models to separate the behaviors and symptoms of multiple anxiety disorders and PTSD. If it can be divided, the novel anxiolytic drugs might be more clearly separated for treating these different anxiety disorders. In summary, the limitations should be considered for further studies.

## 8. Conclusions

The development of anxiolytic drugs’ discovery should be considered for which anxiety disorders and anxiolytic drugs underlined different neural mechanisms, including classical neurotransmitters, neuropeptides, and nonclassical neurotransmitter systems. Moreover, the side effects, neurotoxin, and shortages should be discussed and considered in the clinical trials. The nonclassical neurotransmitters (except for inflammatory cytokines and BDNF) appeared in far fewer published papers. Therefore, this line of studies for nonclassical neurotransmitters calls for further investigation.

The different animal models of anxiety disorders and PTSD have different characteristics, advantages, and disadvantages. We provided some suggestions on how and when to use these animal models for building up or testing anxiety disorders and PTSD. The basic requirements for consideration are face, predictive, and constructive validities. The present review contributes some clinical insights for screening novel anxiolytic drugs.

## Figures and Tables

**Table 1:** Prevalence and symptoms of eight major anxiety disorder types as described in the DSM-5.

Anxiety Disorders	Prevalence	Symptoms
1. Generalized anxiety disorder (GAD)	Approximately 0.9% and 2.9% prevalence rates for adolescents and adults in the United States.	1. Persistent and excessive anxiety. 2. Worry about school and work performance.
2. Panic disorder	Approximately 2–3% for adolescents and adults in the United States.	1. Recurrent unexpected panic attacks. 2. Persistently concerned or worried about further panic attacks.
3. Agoraphobia	Approximately 1.7% for adolescents and adults in the United States.	1. Significant and intense fear or anxiety induced by an extendable range of surroundings in real or anticipated exposure.
4. Post-traumatic stress disorder (PTSD)	Approximately 3.5% for adults in the United States.	1. Concern intrusions and avoidance of memories associated with the traumatic event itself. 2. The critical features of PTSD vary. 3. Some patients encounter fear-based reexperiencing, emotional, and behavioral symptoms. 4. Others feel anhedonic or dysphoric mood states, and negative cognitions may be most distressing. 5. In some cases, arousal and reactive externalizing symptoms are prominent. 6. Others produce dissociative symptoms that predominate. 7. Some individuals exhibit combinations of these symptom patterns.
5. Social anxiety disorder (SAD; social phobia)	Approximately 7% in the United States.	1. Social phobia. 2. Fearful or anxious about or avoidant of social interactions and social surroundings that involve the possibility of being scrutinized.
6. Acute stress disorder (ASD)	Less than 20% (do not involve interpersonal assault) in the United States.	1. Symptoms may vary by individual. 2. Anxiety response for reexperiencing or reactivity to the traumatic event. 3. A dissociative or detached presentation, although these individuals typically will also display strong emotional or physiological reactivity in response to trauma reminders. 4. A strong anger response in which reactivity is characterized by irritable or possibly aggressive responses. 5. The symptoms are development at least lasting from 3 days to 1 month.
7. Separation anxiety disorder	About 0.9–1.9% for adults, 4% for children, and 1.6% for adolescents in the United States.	1. Excessive fear or anxiety concerning separation from home or attachment figures.
8. Obsessive–compulsive disorder (OCD)	About 1.2% in the United States.	1. The presence of obsessions and compulsions. 2. Obsessions are repeated, persistent thoughts, images, or urges. 3. Persistent thoughts are voluntarily associated with marked distress or anxiety. 4. Compulsions are repetitive behaviors or mental acts.

**Table 2:** Pharmacological treatments for eight types of anxiety disorders in the DSM-5.

Anxiety Disorders and Treatments									
**Medicines**	Drugs	1. GAD	2. PD	3. Agoraphobia	4. PTSD	5. SAD	6. ASD	7. Separation Anxiety Disorder	8. OCD
1. BDZs	Alprazolam	V	V	V	V	V	V	V	V
	Chlordiazepoxide	V	V	V	V	V	V	V	V
	Clonazepam		V						
	Diazepam	V	V	V	V	V	V	V	V
	Lorazepam	V	V	V	V	V	V	V	V
	Oxazepam	V	V	V	V	V	V	V	V
2. SSRIs	Escitalopam	V	V	V		V			
	Fluoxetine		V	V					V
	Fluvoxamine		V	V		V			V
	Paroxetine	V	V	V	V	V			V
	Citalopram		V	V		V			
	Sertaline	V	V	V	V	V			V
3. SNRIs	Duloxetine	V							
	Venlafaxine	V	V	V		V			
4. TCA	Clomipramine		V	V					V
	Doxepine	V	V	V	V	V	V	V	V
	Imipramine		V						
5. MAOIs	Phenelzine		V						
	Moclobemide					V			
6. Calcium modulators	Pregabalin	V				V			
7. Azapirone	Buspirone	V	V	V	V	V	V	V	V
8. Antihistamine	Hydroxyzine	V	V	V	V	V	V	V	V

Note: (V) indicates that this drug is used in specific anxiety disorders. *DSM-5: Diagnostic and Statistical Manual of Mental Disorders, Fifth Edition*. Acute stress disorder (ASD); benzodiazepines (BDZs); generalized anxiety disorder (GAD); monoamine oxidase inhibitors (MAOIs); obsessive–compulsive disorder (OCD); panic disorder (PD); post-traumatic stress disorder (PTSD); serotonin-norepinephrine reuptake inhibitors (SNRIs); selective serotonin reuptake inhibitors (SSRIs); social anxiety disorder (SAD); tricyclic antidepressant (TCA).

**Table 4:** Various animal models of anxiety disorders and PTSD in shaping anxiety models and testing anxiety behaviors.

Animal Models	Characteristics	Advantages	Disadvantages	When to Use	Use Frequency	References
A. Shaping anxiety models						
1. Fear conditioning: Cue/footshock	Applying a discrete cue stimulus to pair with footshock-induced stress.	Cue is a clear-cut stimulus with high face, predictive, and constructive validity.	---	Anxiety disorders; PTSD	***	[59]
2. Fear conditioning: Context/footshock	Applying a contextual stimulus to pair with footshock-induced stress.	A contextual stimulus mimics the environment: high face, predictive, and constructive validity.	Context is a complex stimulus combining various environmental stimuli.	Anxiety disorders; PTSD	***	[60]
3. Single prolonged stress	Animals are restrained for 2 h and then forced to swim tests for 20 min. Following recovery for 15 min, animals are exposed to ether until they lose consciousness.	Stable stress; face, predictive, and constructive validity.	Require complex and long-term stress manipulations. The single prolonged stress model is complex compared to the fear conditioning model.	PTSD	***	[61]
4. Learned helplessness	Animals are exposed to uncontrolled stressors through behavioral responses.	Manipulate footshock to shape the stressor; thus, effective and easy manipulation.	Also used to test depression behaviors.	PTSD; MDD	*	[62,63]
5. Restraint stress	Mice are immobilized by placing them into well-ventilated 50 mL Falcon tubes for 2 h per day over 21 consecutive days.	Restraint mice for immobility to induce the stressor; easy preparation and manipulation.	Also used to test depression behaviors.	Anxiety disorders; PTSD	*	[64]
6. Inescapable tail shock	Animals experience uncontrolled and inescapable tail shock, leading to acute stress.	Easy manipulation for inescapable tail shock to induce stress.	Also used to test depression behaviors.	PTSD	*	[65,66]
7. Underwater trauma	Animals are held underwater for 30 s.	Easy manipulation for holding animals underwater to induce stress.	Doubt in the face, predictive, and constructive validity.	PTSD	*	[67]
8. Social isolation	Animals are raised without any companion or environmental enrichment.	Easy manipulation for animals without any companions.	Long-term conduction.	PTSD	**	[13]
9. Social defeat	Animals are exposed to a trained aggressor conspecific for 6 h daily for 5 or 10 days.	Easy manipulation for exposing aggressors inducing stress.	---	PTSD	**	[68]
10. Early life stress	Maternal separation induces trauma events.	Face, predictive, and constructive validity.	Long-term conduction.	PTSD	**	[67]
11. Predator-based stress	Predators or predator-related stimuli (such as predator urine) produce trauma induction.	Place the predator and its related stimuli to induce stress; easy manipulation.	---	PTSD	**	[67]
**B. Testing anxiety behaviors**						
1. Open field test	Tests time spent on crossing trials in the center area of the open field task for anxiety responses.	Face, predictive, and constructive validity.	Competition between locomotion and anxiety behavior.	Multiple anxiety disorders; PTSD	***	[69,70]
2. Elevated zero maze test	The test is conducted in the open arm to indicate the strength of the anxiety responses.	No crossing areas, which enforce animals’ decisions.	Conflicts arise from spending time in open arms and closed arms.	Multiple anxiety disorders; PTSD	**	[68]
3. Elevated plus maze test	The test is conducted in the open arm to indicate the strength of the anxiety responses.	Cross the area to take a rest.	Long-term staying in the cross area between the closed and open arms	Multiple anxiety disorders; PTSD	***	[69]
4. Elevated x-maze test	Tests the open arm time/total time ratio.	Face, predictive, and constructive validity.	---	Multiple anxiety disorders	*	[71,72]
5. Light–dark box test	Tests activity and time spent in both brightly lit and dark apparatus compartments using the animal’s innate desire to explore novel areas.	Assessing the activity and time in light and dark boxes; easy manipulation.	---	Multiple anxiety disorders	**	[69]
6. Startle response test	Pairing a conditioned stimulus (sound or light) with a footshock induces an anxiogenic “startle” response.	Face, predictive, and constructive validity for anxiety disorders.	Limitations in the style of anxiety behaviors for a cue with footshock.	Multiple anxiety disorders; PTSD	**	[10]
7. Marble burying test	Animals with previous stress are placed in the test cage and then test amounts of marble burying up to 2/3 of the depth with bedding.	Face, predictive, and constructive validity for anxiety disorders.	A digging activity for a species-typical reaction to stress (e.g., rats and mice).	Multiple anxiety disorders; PTSD	**	[73]
8. Defensive shock-prod burying test	A familiar test cage or home cage with plentiful bedding and a hole in the wall 2 cm above the bedding. An electrical probe is connected to a shock source. Measuring the depth to which the prod is buried.	Face, predictive, and constructive validity.	Animals do not touch the electrical probe and cannot induce anxiety.	Multiple anxiety disorders	**	[74]
9. Grooming test	Stressors (e.g., novel environment, predator exposure, bright light) induce grooming.	Test grooming behavior; simple manipulation.	Questionable face, predictive, and constructive validity.	Multiple anxiety disorders; PTSD	*	[10]
10. Social interaction test	Two mice were in the test environment for 5 or 10 min and recorded the duration and frequency of all social interactions, including sniffing, following, chasing, touching, and biting. Higher scores in social interactions indicate lower anxiety behaviors.	More accessible design and manipulation.	Limitations in social anxiety disorders.	Multiple anxiety disorders; PTSD	**	[10]
11. Suok test	The Suok task simultaneously tests anxiety, vestibular, and neuromuscular deficits by combining an unstable rod with novelty. The threats of height, loss of balance, and novelty are presented to analyze anxiety and assess animal exploration.	Face validity.	Doubt in predictive and constructive validity. Competitions in testing for multiple behaviors.	Multiple anxiety disorders; PTSD	*	[75]
12. Stress-induced hyperthermia test	Based on the evolutionarily important role of hyperthermia, whereby body temperature rises upon encountering stressful stimuli.	Across many species, including humans.	Testing errors from a lot of confounding factors.	Multiple anxiety disorders; PTSD	*	[10]
13. Hole–board test	Tests head-dipping behaviors. More head dips indicate more explorations and lower anxiety.	Assessing animals’ head-dipping behavior; easy preparation and manipulation.	Doubt in the face, predictive, and constructive validity.	Multiple anxiety disorders	*	[76]
14. Rat exposure test	Uses animals’ natural defensive “avoidance” behavioral response to signs of potential danger, such as a natural predator. Defensive behaviors include stretch-attend posture, stretch approach, freezing, burying, and hiding.	Testing nature defensive behavior; thus, it is easy to use and manipulate.	Variations among different species.	Multiple anxiety disorders	*	[10]
15. Novel object test	Testing the approach-avoidance behaviors of mice in response to novel stimuli. Longer time in exploration for a novel object, indicating lower anxiety behaviors.	Face, predictive, and constructive validity.	Confused with recognition tests using the same task.	Multiple anxiety disorders; PTSD	*	[77]

Note: (*) numbers indicated the specific animal models’ usage frequency for anxiety disorders and PTSD; asterisks (*, **, ***) indicate relative usage frequency; (---) indicates no disadvantages.

**Table 5:** Choosing the best animal models for testing a variety of anxiety disorders and clinical drugs.

Animal Models of Anxiety Disorders												Clinical Anxiolytic Drugs
Anxiety disorders	1. Fear conditioning (cue)	2. Fear conditioning (context)	3. SPS	4. Learned helplessness	5. Restraint stress	6. Inescapable tail shock	7. Underwater trauma	8. Social isolation	9. Social defeat	10. Early life stress	11. Predator-based stress	Medicines
1. GAD	V			V								BDZs; SSRIs; SNRIs; TCAs; calcium modulators; azapirones; antihistamines
2. PD		V				V	V					BDZs; SSRIs; SNRIs; TCAs; MAOIs; azapirones; antihistamines
3. Agoraphobia		V										BDZs; SSRIs; SNRIs; TCAs; azapirones; antihistamines
4. PTSD	V	V	V	V	V	V	V	V	V	V	V	BDZs; SSRIs; SNRIs; TCAs; azapirones; antihistamines
5. SAD								V	V			BDZs; SSRIs; SNRIs; TCAs; MAOIs; calcium modulators; azapirones; antihistamines
6. ASD	V	V			V	V					V	BDZs; TCAs; azapirones; antihistamines
7. Separation anxiety disorder								V		V		BDZs; TCAs; azapirones; antihistamines
8. OCD	V			V		V					V	BDZs; SSRIs; TCAs; azapirones; antihistamines

Note: (V) indicates that this drug is used in specific anxiety disorders. *DSM-5: Diagnostic and Statistical Manual of Mental Disorders, Fifth Edition*. Acute stress disorder (ASD); benzodiazepines (BDZs); generalized anxiety disorder (GAD); monoamine oxidase inhibitors (MAOIs); obsessive–compulsive disorder (OCD); panic disorder (PD); post-traumatic stress disorder (PTSD); serotonin-norepinephrine reuptake inhibitors (SNRIs); selective serotonin reuptake inhibitors (SSRIs); social anxiety disorder (SAD); tricyclic antidepressant (TCA).
